# The principles of person‐centredness in quality patient care–Evaluation of the Community Pharmacy Services Quality Guidelines in Estonia

**DOI:** 10.1002/hpm.3567

**Published:** 2022-08-26

**Authors:** Kristiina Sepp, Afonso Cavaco, Daisy Volmer

**Affiliations:** ^1^ University of Tartu Tartu Estonia; ^2^ University of Lisbon Lisboa Portugal

**Keywords:** community pharmacy, healthcare quality, patient care, person‐centred care, person‐centredness, pharmaceutical care

## Abstract

**Introduction:**

Person‐centredness is considered a key component of quality healthcare and the core competence of all healthcare professionals. However, person‐centred care (PCC) is not often considered a priority for improving the quality of healthcare. This study aimed to evaluate to what extent the PCC principles are included in the Community Pharmacy Services Quality Guidelines (CPSQG) in Estonia.

**Methods:**

The deductive content analysis was performed using the PCC framework developed by Santana et al.

**Results:**

Approximately 2/3 (*n* = 78) of the CPSQG indicators (*n* = 126) in the practical guide used in Estonian community pharmacies support PCC principles. These results demonstrate that quality service itself includes some PCC components, as it forms an integral part of quality care and is directly related to its development. More than half (61.6%) of the CPSQG indicators were divided into process (covering the interaction of pharmacists and patients), one fourth into structure (mainly represented as environment and operation topics), and one tenth into outcome category (access to care). This result is in line with the situation of pharmacies in Estonia, where the current focus is on developing and implementing quality services (e.g., quality guidelines, e‐tools supporting dispensing, restructuring of counselling area for private consultations) and finding the necessary resources for described activities.

**Conclusions:**

To support a more effective application of PCC principles in the community pharmacy practice, the CPSQG should be supplemented with indicators identifying patients' individual preferences, values, and needs. Additionally, interactions with other healthcare professionals should be encouraged, and they should be engaged in developing the CPSQG.

## Highlights

- Person‐centredness is an integral part of high‐quality pharmacy service and is directly related to its development.
- Person‐centred care framework can be successfully applied for the evaluation of the practical quality development guideline for community pharmacy setting.
- In order to place a more specific emphasis on person‐centred care, it is necessary to supplement the service guidelines with additional indicators assessing person‐centredness.
- Patients and various healthcare stakeholders should be involved in developing quality guidelines for community pharmacies.

## 1 INTRODUCTION

### 1.1 *“*One size does not fit all.”

Within past decades, patient involvement in healthcare has significantly changed. People's perceptions and norms of health behaviour are influenced by the neoliberal value system, emphasising the individual commitment to health outcomes.^1^ With increased patient involvement in treatment decisions, healthcare professionals also have to follow the principles of person‐centred care (PCC).^2^ According to the Health Foundation, “person‐centred care incorporates the use of clinical skills, evidence‐based knowledge and patient perspective to provide personalised, coordinated care which enables people to make the most of their lives”. Healthcare stakeholders are encouraged to partner with patients and their relatives to create and deliver personalised care.^3^ Furthermore, PCC has also become relevant in improving healthcare efficiency and addressing patient safety issues.^4^, ^5^

### 1.2 Community pharmacists in person‐centred care

Community pharmacists are well‐positioned to take a considerable role in implementing PCC. Pharmaceutical care also covers aspects of PCC, and the lack or absence of the latter significantly affects the quality of the overall service.^6^, ^7^ Thereby, respectfulness, empathy, honesty, and integrity are important to practice as a professional pharmacist.^6^ The Picker Institute outlined that one of the major principles of person‐centred professionalism was to provide adequate and sufficient information to the patient to make choices and for the professionals to respect these choices.^8^ Patients' active participation in the care process and tailoring advice to their needs, combined with individual professional competence, are the most important characteristics of person‐centred professionalism.^9^ PCC consultation in a community pharmacy should promote mutual understanding and non‐judgemental discussions to overcome the gap between the expectations of patients and pharmacists. While patients stress the relevance of emotional aspects of the consultation, pharmacists emphazise the significance of evidence‐based information.^10^

### 1.3 Guidelines of person‐centred care

Good Pharmacy Practice, developed by World Health Organization (WHO) in 1999, and other international and national guidelines and standards describe how pharmacists perform on the best professional level.^11^, ^12^, ^13^, ^14^, ^15^ Traditionally, the PCC concept has not been integrated into guidelines, although WHO consider person‐centredness as a key component of healthcare quality and core competence of all healthcare professionals.^16^ According to Gyllensten et al., most documents about pharmaceutical care focus on resolving pharmacotherapy problems by increasing productivity and efficiency of used resources, but less attention has been paid to PCC.^17^ The heterogeneity of PCC measures and the need to continue developing and implementing person‐centred quality indicators has also been outlined.^18^ It is clear that adherence to the person‐centred concept requires significant changes in existing practices and roles in the healthcare system,^19^ and there are still more evangelists of PCC than actual practitioners.^20^

Various other studies have stressed the need to describe more transparent practices of community pharmacies in providing PCC by involving multiple stakeholders.^17^, ^19^, ^21^, ^22^ In the framework developed by Santana et al., the Donabedian model has been used to structure PCC implementation in healthcare. Here, several “external indicators”, such as organisational characteristics, health information technology, employees' commitment, etc., have been described to demonstrate the multivariate nature of PCC.^19^

This study aimed to evaluate the presence of the PCC principles in the Community Pharmacy Services Quality Guidelines in Estonia.

## 2 METHODS

### 2.1 Study context

At the beginning of 2021, 479 community pharmacies were operating in Estonia, with an average of 2700 inhabitants per pharmacy, similar to other Eastern European countries.^23^, ^24^, ^25^ Community pharmacies in Estonia have historically focussed on traditional services such as compounding and dispensing medicines and providing drug information.^26^ Existing extended services comprise disease prevention, for example, vaccination, smoking cessation, and point‐of‐care testing (e.g., measuring blood pressure and cholesterol).^27^, ^28^, ^29^ Medication use review was first piloted in 2019.^30^ The Medicinal Products Act and other legislation on medicinal products in Estonia contain requirements for handling medicines in community pharmacies, but there is no detailed description for providing quality pharmacy services and contemporary PCC.^31^

A national and voluntary Community Pharmacy Services Quality Guidelines (CPSQG) in Estonia were developed in collaboration with practicing pharmacists and professional organizations, State Agency of Medicines and educational institutions for example, Institute of Pharmacy of the University of Tartu to formulate the principles of current pharmacy services, that is, the quality aspects of services, including quality criteria for service provision (published in 2012 and updated in 2016 and 2021).^32^ The CPSQG print version was distributed to all Estonian community pharmacies in 2012, 2016, and 2021, whereas the electronic version of the CPSQG is available on the web pages of professional organizations for example, Pharmaceutical Society of Estonia, The Estonian Pharmacists' Association.^33^, ^34^ Several introductory workshops were held from 2012 to 2019 to raise awareness of the CPSQG and improve its implementation.^35^ The majority of pharmacists considered CPSQG important; however, lack of motivation and/or time were deemed the main barriers to implementation.^36^

Community Pharmacy Services Quality Guidelines indicators enable pharmacists' self‐assessment of service quality and different operational aspects of community pharmacies but do not have direct indicators to evaluate PCC.^32^ A similar quality guide is also available for general practitioners (GPs), allowing self‐evaluation of their daily practice and asking for patient feedback about GPs' practice performance and general satisfaction with the given service, for example, service accessibility and communication.^37^

### 2.2 Study design and instruments

The CPSQG indicators (*n* = 126) have been used as a self‐assessment tool for community pharmacists in Estonia to evaluate the change in service quality in 2014, 2016, and 2019 (Table 1).^27^ The self‐assessment tool is divided into themes and subthemes with a two‐point (1 yes/0 no) or a four‐point (0 never/1 occasionally/2 mostly/3 always) response scale. To identify PCC principles in the CPSQG, a model developed by Santana was applied to the community pharmacy setting. The framework is based on a narrative review of the PCC literature, and for classification, the Donabedian model for healthcare improvement with structure, process, and outcome categories was used (Table 2).^19^

**TABLE 1:** Indicators used in the Community Pharmacy Services Quality Guidelines (*n* = 126)^26^

Themes (*n* = 3)	Sub‐themes (*n* = 10)	Quality items (*n* = 126)
Traditional community pharmacy services	Prescription‐only medicines (POM)	Prescription check (3)
		Selection of medicines (7)
		Patient counselling on the use of POMs (14)
	Self‐treatment and non‐prescription medicines and other pharmacy goods	Evaluation of symptoms (6)
		Selection of treatment method (6)
		Patient counselling on the use of OTCs or other pharmacy goods (9)
	Compounding of medicines	Handling of prescriptions for extemporaneous medicines (4)
		Preparation of medicines (3)
		Quality of extemporaneous medicines (3)
Extended services	Health promotion	Qualification of pharmacists for provision of extended services (5)
		Provided extended services (5)
Pharmacy environment and operation	Premises and technical equipment of the pharmacy	Conditions for private and patient‐centred counselling (7)
		Service provision supporting tools (3)
	Handling of medicines and pharmaceutical goods	Procurement and ensuring stock (3)
		Storage and dispensing (5)
		Quality problems/management (4)
	Pharmacy management	Management of customer relations (6)
		Personnel management (2)
		Manager's responsibilities (4)
	Communication	Internal communication (3)
		External communication (7)
		Communication obligation (2)
		Pharmacist as a lecturer and author of articles (1)
	Pharmacists' training	Pharmacists' lifelong learning (6)
		Pharmacy as a traineeship institution (4)
	Legal requirements	Compliance with legal requirements (4)

**TABLE 2:** Framework for person‐centred care (PCC)^19^

Structure	Process	Outcome
S1. Creating a PCC culture	P1. Cultivating communication	O1. Access to care
S2. Co‐designing the development and implementation of educational programs	P2. Respectful and compassionate care	O2. Patient‐reported outcomes (PROs)
S3. Co‐designing the development and implementation of health promotion and prevention programs	P3. Engaging patients in managing their care	
S4. Supporting a workforce committed to PCC	P4. Integration of care	
S5. Providing a supportive and accommodating PCC environment		
S6. Developing and integrating structures to support health information technology		
S7. Creating structures to measure and monitor PCC		

### 2.3 Data analysis

Deductive qualitative content analysis was used^38^ to apply the PCC framework by Santana to identify PCC principles in the CPSQG. To increase the comprehensibility and trustworthiness, the research was performed independently by three researchers from Estonia (*n* = 2) and Portugal (*n* = 1).

Firstly the CPSQG quality indicators were coded under the corresponding PCC categories (structure, process, outcome) and domains (e.g., S 1. Creating a PCC culture).^39^ Secondly, after individual structuring of CPSQG indicators according to the PCC framework by Santana, the three researchers followed constant comparison and iterative steps to compare the results in two rounds^40^ and developed the final version of the CPSQG indicators in the PCC domains.

## 3 RESULTS

**FIGURE 1:**
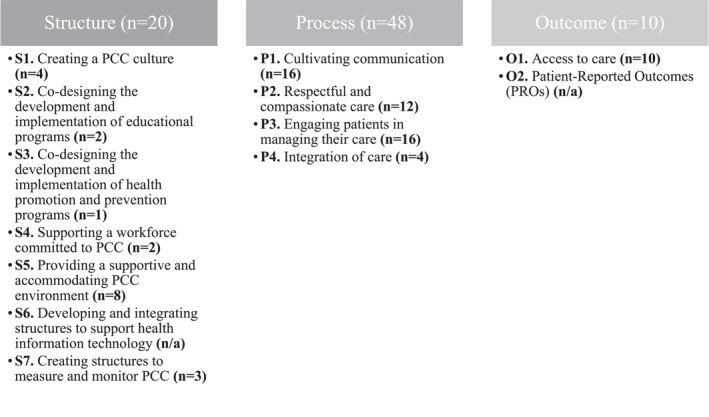
Classification of the Community Pharmacy Services Quality Guidelines quality indicators within person‐centred care (PCC) framework model

The findings demonstrate that 61.9% (*n* = 78) of the CPSQG quality indicators (*N* = 126) support the PCC concept (Figure 1). More than half (61.6%) of these indicators are process‐based, contributing to patient and healthcare provider interaction. The structure category (25.6%) includes PCC domains related to the healthcare system or the care context. The outcome‐based category consists of only 12.8% of the CPSQG indicators.

The CPSQG indicators that did not support the PCC concept (38.1%, *n* = 48) were related to other areas of pharmacy activities such as pharmacy internship, pharmacy equipment, etc.

### 3.1 Structure category

**FIGURE 2:**
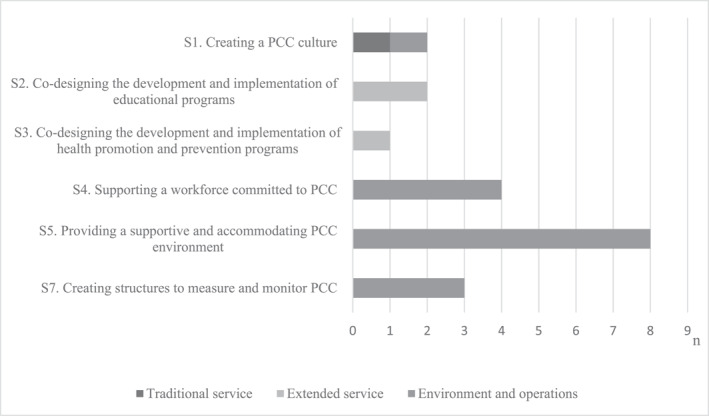
Representation of person‐centred care (PCC) structural domains (n) in the Community Pharmacy Services Quality Guidelines quality indicators

Among the CPSQG indicators, it was possible to identify various structural domains (Figure 2). Providing a supportive and accommodating PCC environment (domain S5, *n* = 8) were the most represented domains, for example, the pharmacy has a separate room for counselling, or the pharmacy has a sufficient number of employees to ensure a quality pharmacy service including detailed counselling. Person‐centred care domains for the development and implementation of educational (S2, *n* = 2) and health promotion and prevention programs (S3, *n* = 1) were covered only under the CPSQG theme of extended community pharmacy services. A limited number of domains was also identified for measuring and monitoring PCC (S7, *n* = 3), for example, during the past 3 years, the pharmacy has carried out a satisfaction survey among customers. Implementing the PCC framework emphasises the importance of the environment and work organisation in creating a service based on the patient's needs.

The complete list of CPSQG indicators categorised according to the PCC structural domains can be found in Appendix (Table A1).

### 3.2 Process category

**FIGURE 3:**
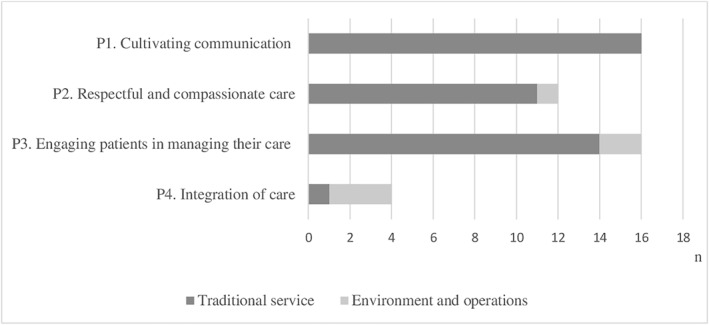
Representation of person‐centred care (PCC) process domains (n) in the Community Pharmacy Services Quality Guidelines quality indicators

Many of the CPSQG indicators of the traditional services (e.g., counselling about prescription and non‐prescription medicines) were classified as PCC process‐based domains amongst P1 ‐ cultivating communication–and P3 ‐ engaging patients in managing their care–were the most represented (*n* = 16) (Figure 3). The P1 domain comprised aspects such as the effect of the medicine/treatment method and the specifics of administration details of medicines. The domain P3 includes factors that directly impact patient outcomes through shared decision making and support, for example, asking about aspects of the patient's lifestyle, general condition, and other factors that may affect treatment outcomes.

Effective communication requires respectful and compassionate care (P2), described in 12 CPSQG indicators, for example, while counselling, the pharmacist makes sure the patient remembers the most critical aspects of the information given.

Only four of the CPSQG indicators enabled to assess indirectly or directly cooperation with other healthcare professionals (domain P4 ‐ integration of care), for example, asking the patient to contact a physician if the symptoms do not improve; if substance abuse or poor medication adherence can be suspected, the pharmacist contacts the doctor, nurse, or social worker.

The complete list of CPSQG indicators categorised into the PCC process domains can be found in Appendix (Table A2).

### 3.3 Outcome category

Access to care (O1, *n* = 10) was the only outcome domain represented among CPSQG indicators (Table 3), mostly about extended services: point‐of‐care testing, that is, blood pressure, cholesterol, blood glucose, haemoglobin, and weight management, and measuring health indicators outside the pharmacy to improve patients' access to care. The availability of medicines also impacts the treatment outcomes. Community Pharmacy Services Quality Guidelines quality indicators, such as the extemporaneous medicine being prepared within 48 h or if the prescribed/asked medicine is out of stock, the pharmacist orders the medicine to the pharmacy or sends the patient to another pharmacy, measure factors influencing the medication patient health outcomes. Also, financial aspects can impact access to care–pharmacists are obliged to offer the cheapest medicine on the reimbursement list to the patient.

**TABLE 3:** Quality indicators of the Community Pharmacy Services Quality Guidelines that belong to access to care domain of person‐centred care (PCC) outcome category by sub‐themes and themes

PCC domain	Quality indicator	Sub‐theme	Theme
O1. Access to care (n = 10)	The cheapest medicine is recommended to the patient.	Selection of medicines	Traditional pharmacy services
	If the requested medicine is out of stock; the pharmacist orders the medicine or sends the patient to another pharmacy where medicine is available, having agreed on that with the other pharmacy beforehand.		
	The cheapest/in different price medicinal product within the active substance is informed.	Selection of treatment method	
	The medicine is prepared within 48 h from submitting the prescription.	Handling of prescriptions for extemporaneous medicines	
	During the past 2 years, pharmacists have offered to measure health indicators outside the pharmacy.	Qualification of employees and provision of extended services	Extended pharmacy services
	Blood pressure is measured.	Provided extended services	
	Blood sugar level is measured.		
	Total cholesterol level in blood is measured.		
	Haemoglobin level in blood is measured.		
	Body composition is analysed.		

## 4 DISCUSSION

This is the first study in Estonia to identify PCC principles in a practical guide–Community Pharmacy Services Quality Guideline. During the development of the CPSQG, the focus was set on quality pharmacy practice, including the development and implementation of different pharmacy services. The CPSQG has enabled detailed feedback on various aspects of community pharmacy services (e.g., compounding and dispensing of medicines, provision of extended services) from the service provider's point of view.^27^ This study focussed on whether the CPSQG indicators can evaluate the PCC principles in community pharmacy practice.

In this study, a framework developed by Santana et al. for introducing PCC in healthcare was applied.^19^ According to the authors' knowledge, this is the first time this framework was used to evaluate a practical guideline.^41^ Instead of building a new guideline, the authors evaluated the content of an existing CPSQG on PCC. It also provided an opportunity to assess how a general theoretical framework can be applied to examine the content of a practical assessment tool. Based on the authors' experience, the framework is generalisable and well adaptable to a community pharmacy setting.

The content analysis demonstrated that 2/3 of the CPSQG indicators support the PCC concept, being most frequently (61.6%) classified in the process category, followed by the structure (25.6%) and outcome (12.8%) categories. The result also correlates with the design of the CPSQG. It reflects the current situation of community pharmacies in Estonia: the main emphasis is set on developing, and standardisation the quality of different services, for example, processes, various structural measures to support pharmacy activities are gradually being supplemented, but until now, little attention has been paid to outcomes of community pharmacy services.^27^, ^29^, ^42^ This is also confirmed by the results of this study, where only those CPSQG indicators that describe access to the service can be added to the outcome category in the PCC framework, but no indicator can be used to measure patient‐reported outcomes.

The bottlenecks related to the outcomes of the pharmacy services, such as low integration into primary healthcare, lack of workforce, and fragmentation of the pharmacy sector, have also been described in other studies.^27^, ^42^ As the contact with the patient does not have to be regular to provide the traditional services and the corresponding extended services are not yet implemented in Estonia, it is not possible in the current situation to assess the outcomes of the pharmacy services for the patient's health or quality of life. A similar situation has been described in other studies.^11^, ^14^, ^15^ It is needed to develop and integrate structures to support health information technology through a continuum of care. Access to data could be seen as one barrier to creating outcome indicators.

The more effective integration of pharmacies into the healthcare system could be one solution to overcome practice issues. Several studies have shown that increased collaboration between pharmacists and other healthcare professionals improves patient health outcomes, access to care and increases the satisfaction of patients and pharmacists.^43^, ^44^ In this study, the PCC process domain “Integration for care” was poorly covered by CPSQG as only four indicators measure this activity. This describes well the current pharmacy position and role within the healthcare system in Estonia ‐ the integration of pharmacy services into primary healthcare has been insufficient, and the society has not used the full professional potential of pharmacists.^42^ In addition, community pharmacy service is not legally qualified as a healthcare service nor involved in providing primary healthcare services.^45^ When developing a tool for PCC and service quality evaluation, different stakeholders should be engaged to meet their needs. The community pharmacy sector must reinforce its positioning in the healthcare chain.^42^, ^46^ Adopting a collaborative approach improves patient health outcomes, and pharmacists can also ensure that care is integrated across health care and for all stakeholders.^17^, ^47^, ^48^

Outcomes are directly linked to the processes leading to the expected results.^49^ For example, patient counselling on medicines usage improves adherence, patient health outcomes, and general patients' satisfaction with services.^50^, ^51^, ^52^, ^53^, ^54^, ^55^ This study identified that most of the CPSQG indicators of medicines counselling fit well into the process category of the PCC framework. The indicators are designed to consider patients' individual preferences, values, and needs to increase their independence and autonomy. However, the three pillars of person‐centred communication ‐ openness, active listening, and plain‐speaking, should also be integrated into the guidelines to better engage the patient in their care.^56^, ^57^ King's Patient‐Centred Communication Tools can assist pharmacists in developing their person‐centred communication skills.^58^ Person‐centred communication is an important element in building a solid interpersonal relationship with patients. It is up to the pharmacist to frame the conversation with the patient and improve the medication experience for the patient, which is also an important component in cultivating pharmaceutical care practice and PCC.^59^ Still, PCC is not about giving patients whatever they want, or providing information only; it is co‐production to develop appropriate solutions together.^60^ As the CPSQG quality indicators have been created only by pharmacists, and the focus has been on improving the quality of provided services, it is essential to engage patients in the development process of CPSQG and foster PCC in a community pharmacy setting. Patient input is critical in improving aspects of communication, which is a weakness of the CPSQG. Patients can provide information that professionals may not know, and more substantial involvement shapes the delivery and quality of care.^61^ When supplementing the CPSQG in the future, it is possible to follow the quality guideline of GPs, which also includes instructions for receiving patient feedback.^37^

The PCC structure category embodies the necessary healthcare resources (e.g., people, tools, systems), in the context of providing care and covering organisational characteristics. The pharmacy environment should support PCC and ensure meaningful interactions with patients. Lack of privacy is considered the main obstacle for patients to open up and freely speak about their health problems.^62^ Currently, the pharmacy environment could be more supportive of private consultations in Estonia. The CPSQG‐based self‐assessment in 2019 showed that only 16% of community pharmacies have a separate counselling room at the pharmacy.^27^

Furthermore, it is crucial having adequate resources to practice PCC (domain S4). The development of a PCC service is complicated if the financing of the pharmacy services is product‐based^31^ and the average retail pharmacy mark‐up is low (in Estonia 13.1% EUR).^63^ New reimbursement schemes, for example, value‐based healthcare that focuses primarily on improving patient health outcomes, supporting caregivers' professionalism, working in teams, and providing care, align with how patients experience their health.^64^ In addition, having a sufficient number of pharmacists to promote and practice PCC is crucial. Recent CPSQG‐based self‐assessments have shown an increasing problem with a limited workforce.^27^

Also, continuing education and following the principles of life‐long learning is vital to ensure pharmacists' professionalism and cultivate PCC. Several CPSQG indicators support the continuous education of pharmacists, but there is still a need to develop an integrated continuing education system in Estonia, including the assessment of professional knowledge and skills of pharmacists.^65^ Systematised continuing professional education is necessary to facilitate the implementation of the PCC concept in the pharmacy setting. However, without a professional culture that appreciates patients' viewpoints, there will be limited drive to develop educative programs that follow PCC principles.^19^

### 4.1 Limitations

The conceptual framework used in this study is a tool for providing PCC in healthcare organizations. Although it is well adaptable for a community pharmacy setting, not all sector‐specific features supporting PCC may be considered.

The framework is considered more as a roadmap for the PCC implementation, but in this study, it was used for retrospective assessment for identification of PCC principles in already developed and, to some extent, implemented guidelines (Community Pharmacy Services Quality Guidelines).

The classification of the indicators into PCC domains may have been influenced by the evaluators' expertise and practical experience. The result obtained should be validated by a more significant number of evaluators.

## 5 CONCLUSION

Community Pharmacy Services Quality Guidelines is a well‐designed tool focussing on the quality development of community pharmacy services in Estonia and supporting PCC principles. The analysis was performed using the PCC framework by Santana et al., which can also be successfully applied for evaluating the content of an existing guideline for a community pharmacy setting. More than half of the CPSQG indicators were divided into process, one fourth into structure, and one tenth into outcome category. This result is in line with the current situation of pharmacies in Estonia, where the focus is on developing and implementing of services and finding the necessary resources for described activities.

To support a more efficient application of PCC principles in the community pharmacy practice, the CPSQG should be supplemented with indicators identifying patients' individual preferences, values and needs. Some of the existing indicators should be re‐written to reach a truly patient‐centred approach. Patients and various healthcare stakeholders should be involved in developing the guide, as PCC is a co‐creation for suitable solutions. Further studies should address the impact of implementing CPSQG on PCC.

## CONFLICT OF INTEREST

The author declares that there is no conflict of interest that could be perceived as prejudicing the impartiality of the research reported.

## ETHICS STATEMENT

Not applicable.

## Supporting information

Supplementary Material

## Data Availability

The data that supports the findings of this study are available in the supplementary material of this article.
