# Supplementary material for: The principles of person‐centredness in quality patient care–Evaluation of the Community Pharmacy Services Quality Guidelines in Estonia
Source: Int J Health Plann Manage. 2022 Aug 26;37(Suppl 1):101–14. doi: 10.1002/hpm.3567 (PMC10086785; doi:10.1002/hpm.3567)
Supplement: Supplementary file 1 — Supplementary Material [file HPM-37-101-s001.docx]

**Appendix**

**Table A1.** Quality indicators of the Community Pharmacy Services Quality Guidelines (CPSQG) categorised according to the person-centred care (PCC) framework to structure category by sub-themes and themes

| **PCC domain** | **CPSQG quality indicator** | **Sub-Themes** | **Themes** |
| --- | --- | --- | --- |
| S1. Creating a PCC culture (n=4) | A pharmacy that is not obliged to prepare medicines & arranges the medicine preparation at another pharmacy. | Handling of prescriptions for extemporaneous medicines | Traditional pharmacy services |
|  | To improve work organisation, the pharmacy manager asks for feedback and suggestions from all employees at least once a year. | Management of customer relations | Environment and operation |
|  | The pharmacy has approved the principles of personnel management listed in CPSQG | Personnel management |  |
|  | If a customer needs to be sent to another pharmacy for a medicine, the pharmacist first agrees with the other pharmacy. | External Communication |  |
| S2. Co-designing the development and implementation of educational programs (n=2) | All staff who provide the extended health promotion and illness prevention service have completed the required training and have a corresponding certificate. | Qualification of employees and provision of extended services | Extended pharmacy service |
|  | All staff who measure health indicators have completed training for this purpose and have a corresponding certificate. |  |  |
| S3. Co-designing the development and implementation of health promotion and prevention programs (n=1) | During the past two years, the pharmacy has taken part in health and environmental campaigns – directed to and developed in collaboration with respective patients groups | Qualification of employees and provision of extended services | Extended pharmacy service |
| S4. Supporting a workforce committed to PCC (n=2) | All employees of the pharmacy are aware of and follow unified principles of customer service and problem-solving | Management of customer relations | Environment and operation |
|  | All pharmacy employees engaged in customer service are proficient in Estonian. |  |  |
| S5. Providing a supportive and accommodating PCC environment (n=8) | Private counselling while dispensing prescription medicines is ensured. | Conditions for private and patient-centred counselling | Environment and operation |
|  | Private counselling while dispensing OTC medicines is ensured. |  |  |
|  | The pharmacy has a separate room for counselling. |  |  |
|  | The customer has a place to sit while a prescription medicine is dispensed. |  |  |
|  | The customer has a place to sit while an OTC medicine is dispensed. |  |  |
|  | The sales room has seats for customers waiting for their turn to be serviced. |  |  |
|  | Drinking water is available at the pharmacy to immediately administer a medicine. |  |  |
|  | The pharmacy has enough employees to ensure quality pharmacy service, including thorough counselling (considering different times of day and month). | Personnel management |  |
| S7.Creating structures to measure and monitor PCC (n=3) | Problems related to the pharmacy service and their solutions are documented at the pharmacy. | Management of customer relations | Environment and operation |
|  | The pharmacy has organised the collection of feedback (incl. complaints and suggestions) from visitors (written, website, e-mail) |  |  |
|  | During the past three years, the pharmacy has carried out a satisfaction survey among its customers |  |  |

**Table A2.** Quality indicators of the Community Pharmacy Services Quality Guidelines (CPSQG) categorised according to the person-centred care (PCC) framework to process category by sub-themes and themes

| **PCC domain** | **CPSQG quality indicator** | **Sub-Themes** | **Themes** |
| --- | --- | --- | --- |
| P1.Cultivating communication(n=16) | The effect of the medicine is explained in a comprehensible manner. | Patient counselling on the use of POMs | Traditional pharmacy services |
|  | The specifics of administration arising from the pharmaceutical form are explained. |  |  |
|  | The more common adverse drug reactions and interactions related to the medicine are explained. |  |  |
|  | The patient is counselled on the use of tools needed to administer the medicine (e.g., insulin needles, syringes, nebulisers, etc.). |  |  |
|  | The rate of medicines action is explained. |  |  |
|  | The correct storage and the expected storage time of the medicine are explained. |  |  |
|  | If the patient has any additional questions about the illness(es) or medicine(s) is asked. |  |  |
|  | Nature of discomfort. | Evaluation of health problem symptoms |  |
|  | Duration of discomfort. |  |  |
|  | Concomitant symptoms. |  |  |
|  | The pharmacist points out important aspects of the medicine and/or product (e.g., taste, form and other use specifics, overdose toxicity, contraindications). | Selection of treatment method |  |
|  | The effect of the medicine/treatment method is explained to the customer in a comprehensible manner. | Patient counselling on the use of OTCs or other pharmacy goods |  |
|  | The administration and dosage of the medicine and other relevant treatment techniques are explained. |  |  |
|  | The duration of the treatment/use of the treatment method is explained. |  |  |
|  | The proper storage of the medicine and the expected in-use storage time are explained. |  |  |
|  | The onset of action of the medicine/treatment method is explained. |  |  |
| P2. Respectful and compassionate care (n=12) | The pharmacist checks whether the medicine has any contraindications in the particular case. | Selection of medicines | Traditional pharmacy services |
|  | The usage guidelines are always written on the medicine package unless the patient asks not to. | Patient counselling on the use of POMs |  |
|  | While counselling, the pharmacist makes sure the patient remembers the most important aspects of the information provided. |  |  |
|  | If the patient’s symptoms are severe or their reason is unclear, the pharmacist advises the patient to see a doctor. | Evaluation of health problem symptoms |  |
|  | What purpose the selected OTC medicines or other pharmacy products are going to be used is assessed. | Selection of treatment method |  |
|  | Factors of customer that may influence the choice and treatment outcome of the OTC medicine or other pharmacy product is identified. |  |  |
|  | Where appropriate, the possibility of non-medical treatment will be suggested |  |  |
|  | The more common adverse drug reactions and interactions related to the medicine/treatment method are explained. | Patient counselling on the use of OTCs or other pharmacy goods |  |
|  | The pharmacist asks whether the customer has any additional questions. |  |  |
|  | While counselling, the pharmacist makes sure the customer remembers the most important aspects of the information. |  |  |
|  | The patient is asked to return to the pharmacy or seek the doctor’s advice if the patient has any problems using medicines. |  |  |
|  | In case of medicines that require additional steps before administration, the pharmacy always offers to prepare the medicine for the customer. | Storage and dispensing | Environment and operation |
| P3. Engaging patients in managing their care (n=16) | Always identify the buyer of the medicine based on the identification document. | Selection of medicines | Traditional pharmacy services |
|  | Find out whether this is the first or repeat prescription for the patient. |  |  |
|  | The compatibility of the pharmaceutical form to the treatment regimen is checked. |  |  |
|  | If the required medicine is missing from the wholesale network, the pharmacist develops procurement. |  |  |
|  | The dose of the medicine remains within the usual limits (considering the patient’s age) is checked. |  |  |
|  | The amount of the prescription medicine complies with the treatment period is assessed. |  |  |
|  | The name of the patient is written on the package of the medicine. | Patient counselling on the use of POMs |  |
|  | The patient is advised to read the information leaflet contained in the package. |  |  |
|  | The aspects of the patient about any lifestyle, general condition and other factors that may affect treatment outcome are asked. |  |  |
|  | Evidence-based additional information about the illness and/or the medicine is given. |  |  |
|  | The patient is asked to return to the pharmacy or seek the doctor’s advice if the patient has any problems using medicines. |  |  |
|  | The person who has a health problem or needs the OTC medicine and/or product is identified | Evaluation of health problem symptoms |  |
|  | Concomitant illnesses and the use of (prescription) medicines are taken into account. |  |  |
|  | What medicines, products, or treatments the patient has used before and what has been the result of their use? | Selection of treatment method |  |
|  | The pharmacist advises the patient (and also the doctor, if necessary) to apply for compensation for the medicine by way of exception | Storage and dispensing | Environment and operation |
|  | If a wrong medicine has been dispensed or this can be suspected, the pharmacist immediately contacts the patient and/or the doctor. | Communication obligation |  |
| P4. Integration of care (n=4) | Prescription is not correctly/fully prepared, and the pharmacist contacts the medicines prescriber. | Checking correction of prescription | Traditional pharmacy services |
|  | The prescriber shall be notified if he/she has mistaken the requirements of the prescription procedure. | External Communication | Environment and operation |
|  | The pharmacy forwards medicine-related relevant information, including that about shortages and cheaper medicines, to its cooperation partners. |  |  |
|  | If substance abuse or poor medication adherence can be suspected, the pharmacist contacts the doctor, nurse, or social worker. |  |  |
